# Whole-genome resequencing of an acute gamma-mutagenised *Oryza sativa*: a genomic data for discovering the landscape of genomic variants related to bacterial leaf blight resistance

**DOI:** 10.1016/j.dib.2026.113323

**Published:** 2026-10-08

**Authors:** Siti Amira Adilah Kamarudin, Nor’Aishah Hasan, Faiz Ahmad, Nurhamimah Zainal Abidin

**Affiliations:** a Faculty of Applied Sciences, Universiti Teknologi MARA, Cawangan Negeri Sembilan, Kampus Kuala Pilah, 72000 Kuala Pilah, Negeri Sembilan, Malaysia; b Agrotechnology and Biosciences Division, Malaysian Nuclear Agency, 43000 Kajang, Selangor, Malaysia

**Keywords:** Agronomic traits, Bacterial leaf blight, Mutagenesis, MR297, Rice

## Abstract

The use of induced gamma irradiation on a widely cultivated commercial rice variety in Peninsular Malaysia, MR297, has led to the emergence of a promising mutant line, ML-1 that exhibits high yield potential and enhanced resistance to bacterial leaf blight disease. To characterise the genomic changes underlying these improved traits, we sequenced the mutant genome on Illumina NovaSeq 6000 at 40X depth and generated 14.39 Gbp of raw nucleotides consisting of 95.95 million paired-end reads with average insert size of 150 bp. These reads were cleaned, trimmed, aligned to a reference genome (AGIS1.0 genome assembly) and deduplicated. We discovered a total of 4013,384 variations across the genome post-mapping where 90.85% of them are single nucleotide polymorphisms (SNPs) and 9.15% are insertion-deletion mutations (InDels). Functional annotation analysis has annotated 10.9 million of variant effects and classified them according to their impact levels. Sequencing data are publicly available at the NCBI Sequence Read Archive repository under the accession number SRR37260596. These datasets provide valuable genomic resources for comparative genomics, mutation breeding and marker development in rice.

Specifications Table

**Table:** 

Subject	Biology
Specific subject area	Rice genomics
Type of data	Raw reads (fastq files), VCF files and variant annotation table.
Data collection	The total genomic DNA was extracted from the leaves of the mutant ML-1 using the Monarch® Genomic DNA Purification Kit and whole-genome sequencing was performed on the Illumina NovaSeq 6000. The sequencing data was quality assessed with FastQC v0.12.1, trimmed with Trimmomatic v0.39, read mapped with BWA-MEM v0.7.17, duplicated reads marked with samtools markdup v1.19.2, variant called with bcftools mpileup v1.19, and functionally annotated with SnpEff v5.2.1.
Data source location	Isolation source: Leaf tissue of rice Institution: Malaysian Nuclear Agency City/Town/Region: Kajang/Selangor Country: Malaysia Latitude and longitude: 2.9108° N, 101.7705° E
Data accessibility	Data is publicly available at the Sequence Read Archive (SRA) repository of the National Center for Biotechnology Information (NCBI): BioProject accession: PRJNA1424657 BioSample accession: SAMN55390484 SRA: SRR37260596 Direct URLs to data: BioProject: https://www.ncbi.nlm.nih.gov/bioproject/PRJNA1424657 BioSample: https://www.ncbi.nlm.nih.gov/biosample/SAMN55390484 SRA: https://www.ncbi.nlm.nih.gov/sra/SRR37260596 Data files and bash scripts used for bioinformatics analyses available at: https://github.com/AmiraKamarudin/Rice-Mutant-Line-1 (v1.1)
Related research article	None

## 1 Value of the Data

• This genomic data enables the development of functional single nucleotide polymorphism (SNP) and insertion/deletion (InDel) markers that would be utilized in marker-assisted selection of successive mutants, hybrids, and segregating populations.
• The functionally characterised variants will facilitate researchers to test the associations between the variants with selected agronomic and disease traits using approaches such as gene expression quantification analysis, genome-wide association study (GWAS), quantitative trait locus (QTL) mapping and genetic linkage analysis.
• The annotation data will aid in leveraging deeper exploration of the altered interplay among the defense mechanisms in mutagenised rice genotypes, and providing greater understanding of mutagenesis-induced enhanced immunity.

## 2 Background

*Oryza sativa* subsp. *indica* (Family Poaceae, Subfamily Oryzoideae, Tribe Oryzeae) is a diploid long-grained rice cultivated across Malaysian regions [1]. MR297, a local rice variety, is one of the most-sought after *indica* rice for its commercially important high-yielding properties. However, its weak immunity limits productivity whenever bacterial leaf blight disease infects and spreads during an outbreak [2]. This challenge had prompted mutation breeding to improve disease resistance through nuclear application. In 2019, MR297 was exposed to acute gamma irradiation treatment and the surviving mutant line (named as mutant line 1, ML-1) was produced [3]. Interestingly, ML-1 consistently showed improved morphoagronomic traits and enhanced resistance to bacterial leaf blight disease (see Table 1) positioning it as a promising line for the industry. Despite the observed phenotypic improvements, the underlying genomic variants remain largely unexplored. Whole-genome resequencing datasets from mutation breeding remain scarce for Malaysian rice germplasm. Public availability of these datasets facilitates comparative genomic analyses, variant discovery, and reproducibility of mutation breeding studies. In this paper, we generated whole genome sequencing data of ML-1 and a closer look was taken on the variation profile and functional annotation across the mutant genome. This genomic data is important to contribute to the understanding of how mutation breeding can create resilient rice variety and to provide genomic resources for future crop improvement and disease resistance studies. The availability of this data enables the accessibility and reproducibility in the field of comparative genomics across the local *indica* rice varieties and contribute to feasible precision breeding in Malaysia.

**Table 1:** Morphoagronomic traits of the mutant ML-1 compared to the wild type MR297 genotype (mean ± SD). Different letters indicate statistically significant differences (p < 0.05) [4].

	MR297	ML-1
Date to flowering (DTF) (day)	76.67 ± 6.47ᵃ	70.00 ± 0.00ᵇ
Date to maturity (DTM) (day)	107.21 ± 7.30ᵃ	100.00 ± 0.00ᵇ
Yield per hectare (YLDHA)	3.96 ± 1.83ᵇ	7.83 ± 3.13ᵃ

## 3 Data Description

We report the genomic data and analysis of the rice line ML-1 sampled from the fifth mutant population post-acute gamma mutagenesis. Upon phenotypic and disease screening of the mutant and wild type in controlled environment, the data were compared and the mutant exhibited improvements in several economically important traits as shown in Tables 1 and Table 2. The raw paired-end sequencing generated forward and reverse sequences and the data are available at the National Center for Biotechnology Information (NCBI) under the BioProject number PRJNA1424657, BioSample number SAMN55390484 and accession number SRR37260596. Sequencing of the 150 bp library generated >95 million paired-end reads representing approximately 14.4 Gbp with an average genome depth of 38.8x across the covered region (Table 2). Overall, around 92.54% of total bases had a Phred quality score of 30 (Q30) or higher, equivalent to 99.9% base call accuracy. These indicate that the sequencing data were of high quality and reliable for variant discovery. Sequences of Illumina-specific P5 and P7 adapters used in the NovaSeq 6000 sequencing-by-synthesis chemistry are listed in Table 4. The mutant ML-1 trimmed reads were aligned to the 385.7 Mbp annotated Nipponbare *Oryza sativa* reference genome (AGIS1.0; NCBI RefSeq Assembly: GCF_034140825.1) [5]. Genome wide variant identification by comparing the mutant ML-1 genome to the reference genome generated a total of 4013,384 variations composed of 3645,997 (90.85%) SNPs and 367,387 (9.15%) InDels. Frequency of variants per all twelve chromosomes are presented in Fig. 1. These identified variants were further classified according to their effect type, genomic region, and predicted impact on protein-coding gene function using the SnpEff annotation toolbox [6]. From Table 5, the predominant variants were annotated as the upstream gene variants (32.58%), downstream gene variants (30.51%) and intergenic variants (22.57%). Among coding variants, 3.32% (365,231) were missense, 1.88% (206,882) were synonymous, and 0.16% (17,887) were stop-gained. These variant effects were also classified according to their impact levels involving “high”, “low”, “moderate”, and “modifier” categories (Table 6). The Bash scripts used in this pipeline, together with mapping statistics, FastQC pre- and post-filtering summaries, VCF files, and SnpEff variant annotation summary are available on GitHub (https://github.com/AmiraKamarudin/Rice-Mutant-Line-1, v1.1)

**Table 2:** Disease scoring of ML-1 and its parent, MR297 for bacterial leaf blight artificial screening [3]. The disease severity was scored following the IRRI Standard Evaluation System (SES) [7].

Genotype	0–9 scale	Disease severity	Host response
ML-1 (mutant)	1	Small brown specks of pin-point size or larger brown specks without sporulating center	Resistant (R)
MR297 (wild-type)	6	Typical susceptible lesions of 3 mm or longer infecting 11–25 % of the leaf area	Moderately Susceptible (MS)

**Table 4:** Oligonucleotide sequences of Illumina adapters used in sequencing library prep.

Adapter	Sequence
P5	AGATCGGAAGAGCACACGTCTGAACTCCAGTCA
P7	AGATCGGAAGAGCGTCGTGTAGGGAAAGAGTGT

**Fig. 1:**
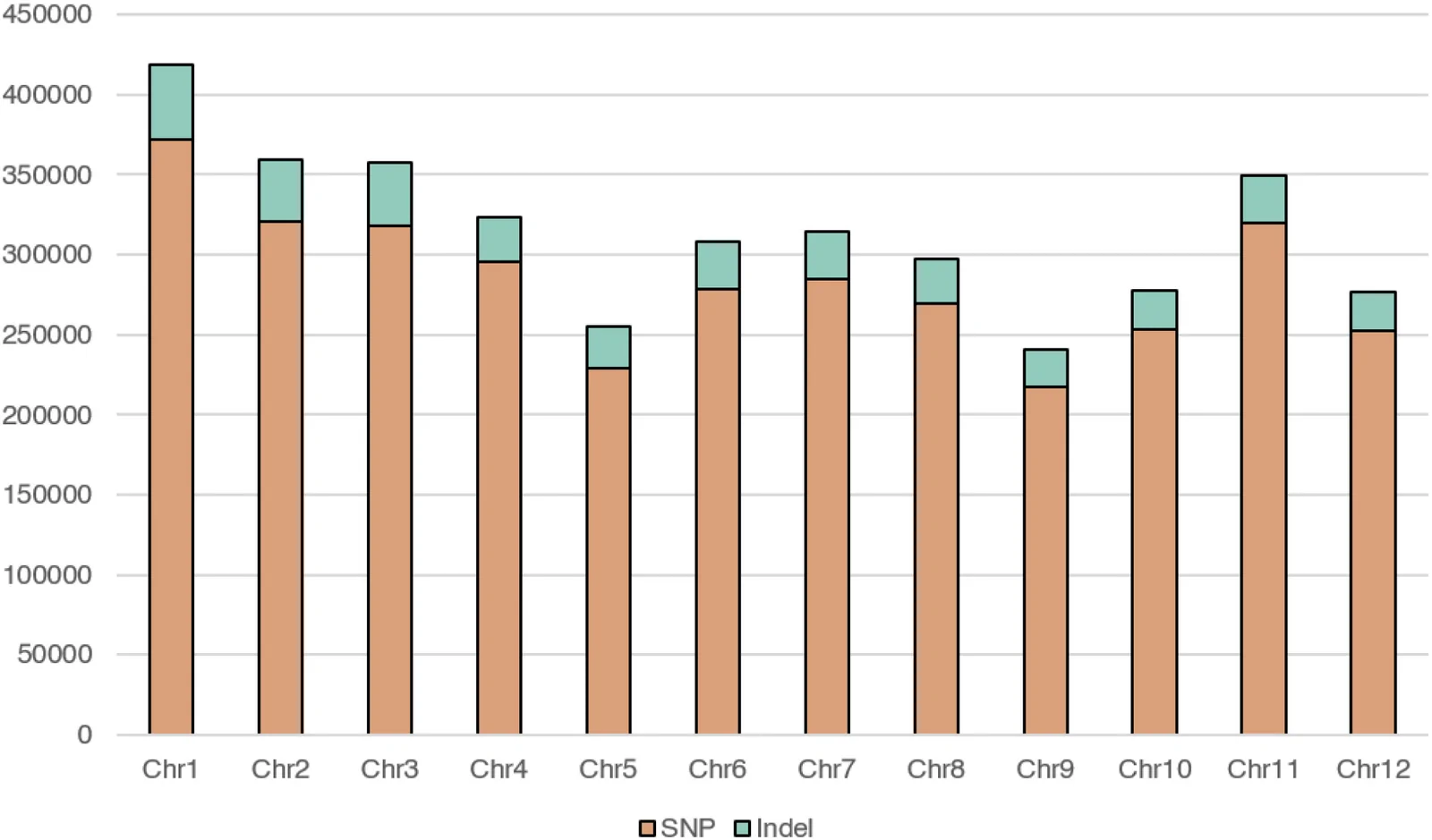
Genome-wide variant frequency in mutant ML-1 genome per chromosome. SNPs: single nucleotide polymorphisms; and InDels: insertions and deletions.

**Table 5:** Counts and percentages of the functional effects of variants on genes predicted and annotated computationally by SnpEff [6]. The effects were categorised by the regions of the effects on the genome. Variants recorded as inputs were SNPs, insertions and deletions. Note that a variant is predicted to impact different transcript isoforms resulting to multiple annotations, hence the total annotation counts exceed the number of variants identified in the genome.

Region	Type	Variant Effect	Count	Proportion (%)
Exon	inframe InDels	Conservative deletion	3435	0.03%
		Conservative insertion	2765	0.02%
		Disruptive deletion	3167	0.03%
		Disruptive insertion	1741	0.01%
	start	Start lost	1340	0.01%
		Start retained	19	0.00%
		Initiator codon	71	0.00%
	stop	Stop lost	9418	0.08%
		Stop gained	17,887	0.16%
		Stop retained	2732	0.02%
	coding	Missense	365,231	3.32%
		Synonymous	206,882	1.88%
		Frameshift	9753	0.09%
		Exon loss	3	0.00%
Untranslated	5’	5’ UTR variant	60,526	0.55%
Region (UTR)		5’ UTR premature start codon gain	6852	0.06%
	3’	3’ UTR variant	97,524	0.89%
Splice	splice	Splice acceptor	2111	0.02%
		Splice donor	2032	0.02%
		Splice region	28,783	0.26%
Intergenic		Intergenic region	2479,231	22.57%
		Upstream gene	3578,964	32.58%
		Downstream gene	3351,804	30.51%
Intron	Intronic	Intron variant	753,496	6.86%
		Intragenic	267	0.00%
		Non-coding transcript	231	0.00%
Total			10,986,265	100%

**Table 6:** Counts and percentages of four impact types of the predicted variant effects on gene functions classified by SnpEff [6]. High-impact effects may indicate truncation or loss-of-function outcomes on proteins, moderate-impact effects are non-disruptive variants that may change protein efficacy, low-impact effects are referred as harmless and have low to no impact on protein functions, and modifiers are non-coding variants where the regions are difficult to predict the impact. High-, moderate- and low-impact flagged variants hit the Coding Sequences (CDS) while modifiers reside outside the coding boundaries.

Effect by Impact	Count	Percentage
High	41,829	0.38%
Low	253,544	2.31%
Moderate	375,256	3.42%
Modifier	10,298,986	93.89%
Total	10,969,615	100%

## 4 Experimental Design, Materials and Methods

### 4.1 Plant material sampling

This study involved comparative profiling of a newly developed mutant rice ML-1 and its wild type MR297. We obtained seeds of both genotypes from Malaysian Nuclear Agency (MNA), Selangor, Malaysia. The seeds were placed in separate petri dishes, surface-sterilised with 80% (w/w) tetramethylthiuram disulfide fungicide solution for 24 h at room temperature and rinsed with distilled water. The pre-germinated seeds were transplanted to soil and grown until 28 days after sowing in the glasshouse. After four weeks, we selected only healthy and lesion-free leaves from the seedlings and collected them using sterilised scissors. The leaves were surface sterilised with 70% ethanol and stored at -20°C for nucleic acid isolation. Plants were grown to maturity to record the morphoagronomic data of both genotypes (see Table 1).

### 4.2 Nucleic acid isolation

The extraction of the genomic DNA of both genotypes was performed using Monarch® Genomic DNA Purification Kit (New England Biolabs, USA) according to the manufacturer’s protocol with minor modification. Tissue disruption step was modified into using Qiagen Tissue Lyser II (Qiagen, Germany) replacing manual vortex mixing. The concentration and purity of gDNA were measured spectrophotometrically using microvolume spectrophotometer (Denovix, DE, USA) and fluorometrically using Qubit™ dsDNA HS Assay Kit (Thermo Fisher Scientific, USA). DNA integrity was checked electrophoretically via 1% (w/v) agarose with FloroSafe DNA stain (First base, Singapore) and ran at 100 V for 60 mins. Only mutant DNA exhibited bands with high integrity and smear-free were selected and sent to the sequencing provider (Azenta Life Sciences, China) for whole genome sequencing.

### 4.3 Whole genome sequencing and data processing

Paired end library of 150 bp insert size was prepared through random gDNA fragmentation, followed by the selection of 150 bp fragments, end repair and dA-tailing, adaptor ligation, PCR amplification, and magnetic bead purification. The mutant library was sequenced to an approximate 40X of average depth with the Illumina NovaSeq™ 6000 platform (Illumina, United States) following the Illumina standard protocol. The sequenced raw reads of mutant genome in FASTQ format were submitted to the Sequence Read Archive (SRA) repository at the National Center of Biotechnology Information (NCBI) under the SRA accession SRR37260596. For comparison of the mutant genome with the wild-type genome, raw paired-end reads of the wild-type, MR297, were retrieved from the ENA database (www.ebi.ac.uk/ena) under the accession number PRJEB32344 **(Zainal Abidin et al., 2018)**. The reads were trimmed with Trimmomatic v0.39 [8] to remove Illumina P5 and P7 adapters **(se**e Table 2), low-quality reads and short reads (<75 bp). The qualities of pre- and post-trimmed reads were checked and compared with FastQC v0.12.1 [9]. The reference genome *O. sativa* Nipponbare (GCF_034140825.1) was indexed with Burrows-Wheeler Aligner (BWA) v0.7.17 [10] and the cleaned reads of mutant and wild-type were assembled and mapped separately to the reference genome using multithreaded bwa mem. We used samtools v1.19.2 [11] to convert, sort the aligned reads from query-sorted to coordinate-sorted, mark and remove duplicates via samtools view, fixmate, markdup, and sort subcommands. The BAM files were used to generate selected alignment metrics as shown in Table 3 with samtools stats.

**Table 3:** Summary of sequencing statistics and read quality of the mutant genome ML-1, raw and post-trim. Q20 and Q30 refer to the proportion of base pairs with Phred-scaled per base accuracy greater or equal to 99% (1 in 100 error rate) and 99.9% (1 in 1000 error rate), respectively.

Name	Raw reads	Trimmed reads	Raw bases (bp)	Trimmed bases (bp)	Q20 (%)	Q30 (%)	GC (%)	Mean depth (X)
ML-1	95,953,586	92,124,845	14,393,037,900	13,818,726,750	97.10	92.54	44.49	38.8

### 4.4 Variant calling

Variant calling against the reference genome was then performed on the BAM outputs using bcftools v1.19 [11] by piping the mpileup, call and filter subcommands altogether to write the single nucleotide polymorphism (SNPs) and insertion/deletion (InDels) identified from mutant and wild type into a single joint VCF file. The VCF was annotated with allelic depth (AD), read depth (DP), genotype quality (GQ), and genotype posterior probabilities (GP). The joint VCF was screened with these following parameters to minimise false positive variants: (i) skip reads with mapping quality ≤ 30 (ii) skip bases with base quality ≤ 20, (iii) keep sites with average mapping quality (MQ) across reads is ≥ 40, (iv) AD ≥ 0.75, DP ≥ 10. The identified variants were visualised in the Integrative Genomics Viewer (IGV) software to validate the nucleotide sequences. All Bash scripts used in this bioinformatics workflow were documented and can be accessed on GitHub (https://github.com/AmiraKamarudin/Rice-Mutant-Line-1, v1.1).

### 4.5 Variant annotation

Functional annotation and effect prediction of the SNPs were performed using SnpEff v5.2.1 [6] with default parameters and a custom-built database. We configured a custom database by creating new genome entry using the Nipponbare genome (GCF_034140825.1) from NCBI and gene annotation file GFF3 retrieved from Rice Super Pan-genome Information Resource Database (RiceSuperPIRdb, http://www.ricesuperpir.com/) under the species accession name T2T-NIP. The variants were assigned to their coding effects and predicted impact on the protein functions based on their genomic position.

## Limitations

Not applicable.

## Ethics Statement

The authors have read the ethical requirements for publication outlined by Data in Brief and declare that the current work does not involve human subjects, animal experiments, or any data collected from social media platforms.

## Credit Author Statement

**Siti Amira Adilah Kamarudin:** Conceptualization, Methodology, Data Curation, Writing – Original Draft. **Nor’Aishah Hasan:** Conceptualization, Supervision, Validation, Writing - Review & Editing, Funding acquisition. **Faiz Ahmad:** Conceptualization, Supervision, Validation, Resources, Writing - Review & Editing, Funding acquisition. **Nurhamimah Zainal Abidin:** Data Curation, Supervision. All authors approved the final manuscript for publication.

## Data Availability

NCBI Sequence Read ArchiveWhole-genome sequencing of Mutant Line 1 (ML-1) (Original data)
